# Patterns of blood cobalt levels after metal-on-metal total hip arthroplasty, predictors of elevated levels, and associations with clinical symptoms

**DOI:** 10.1007/s00264-026-06902-3

**Published:** 2026-07-01

**Authors:** Amanda I. Gonzalez, Nicolas Silvestrini, Cody Fournier, Christophe Barea, Robin Peter, Pierre Hoffmeyer, Didier Hannouche, Anne Lübbeke

**Affiliations:** 1 Division of Orthopaedics and Trauma Surgery, University Hospitals of Geneva, Geneva, Switzerland; 2 Générale-Beaulieu Clinic, Geneva, Switzerland; 3 SWISS Foundation for Innovation and Training in Surgery, Geneva, Switzerland; 4 Nuffield Department of Orthopaedics, Rheumatology and Musculoskeletal Sciences, University of Oxford, Oxford, United Kingdom

**Keywords:** Total hip arthroplasty, Metal-on-metal, Cobalt levels, Head size

## Abstract

**Purpose:**

Objectives were to assess patterns of blood cobalt levels over time in MoM THAs by head size; predictors of elevated cobalt levels; and associations between cobalt levels and clinical outcomes.

**Methods:**

We included all primary MoM THAs performed 1998–2011 with postoperative blood cobalt concentrations. We stratified the MoM THAs in ≤ 36 mm vs. > 36 mm.

**Results:**

Overall, 526 MoM THAs were included: 427 with small and 99 with large heads. Considering patients with at least two serologies before revision (*n* = 205), cobalt levels were higher for patients with large compared to small heads (medians: 3.5 vs. 1.3, *p* < 0.001). We did not find any effect of time on cobalt levels between the two consecutive serologies (*p* > 0.42) or when using time as a continuous variable (*p* > 0.55). For large heads women were more likely to show elevated cobalt levels than men (adjusted OR = 3.56, 95% CI 1.17–10.84, *p* = 0.02). We did not find any significant association between cobalt levels and clinical outcomes (Spearman’s rs < 0.08, ps > 0.15).

**Conclusion:**

We observed stable cobalt levels over time in small and large MoM THAs. Repeat laboratory workups in small and large head MoM THAs seem unnecessary given that the cobalt values remained stable.

## Introduction

Adverse local tissue reactions (ALTRs) due to metal debris and the high revision rates associated with metal-on-metal (MoM) total hip arthroplasty (THA) [1–3] have led authorities and medical societies to develop and regularly update guidelines for the monitoring of patients with these implants [4–7]. Published recommendations include clinical monitoring, blood tests to assess ion levels, and cross-sectional imaging studies [8]. However, a review of the literature on investigation modalities for the surveillance of patients with MoM THA revealed that no single test is sufficient to diagnose failure of MoM THA [8].

There is a decrease in the use MoM bearings [9, 10], but still thousands of patients have a THA with MoM bearing implanted. The surveillance remains particularly challenging when ion level elevation and ALTR are only moderate, and questions remain concerning the long-term surveillance needs of small head MoM THAs, and the association between metal ions levels and clinical outcomes. Furthermore, patient monitoring requires tailored logistics, which also has a cost [11].

After MoM THA, an initial increase in cobalt and chromium blood concentrations is observed during the run-in phase [12], followed by different ion concentration profiles, including a gradual increase in ion levels [13, 14], a plateau followed by a decrease in ion concentration [15, 16], or a plateau followed by an increase [17]. However, all the studies on ion concentration patterns after MoM THA involved only small numbers of patients and primarily concerned resurfacing or large-head THAs.

Predictors of elevated ion levels after MoM THA have been assessed and increased acetabular component inclination [18, 19], younger age [20], and female gender [20, 21] were found to influence ion concentration level in large MoM THA. Less is known about these associations in small head (≤ 36 mm) MoM THA. Finally, the association between clinical scores and blood metal ion levels is poorly studied [22, 23].

Objectives of this study were to assess 1°/patterns of blood cobalt levels over time in MoM THA by head size including after removal of the prosthesis; 2°/predictors of elevated cobalt levels after MoM THAs and according to head size; and 3°/associations between cobalt levels and clinical outcomes (WOMAC pain and function, Harris hip pain, limping, and total score) in patients with MoM THA.

## Patients and methods

### Study design and study population

We included all primary MoM THAs performed between 11/1998 and 03/2011 at the Geneva University Hospitals. All included patients were followed until 31 December 2022. THAs performed for fracture or metastatic disease were excluded. Since March 1996 all patients undergoing THA are prospectively enrolled in the hospital-based arthroplasty registry. Overall, 928 patients met the inclusion criteria, after exclusion of 20 patients who had refused to consent (general consent or registry consent, status 5 June 2023), 1067 primary THAs in 908 patients were included.

With the recognition of issues related to the MoM bearing, blood testing became part of the routine follow-up examination for the monitoring of patients with MoM prostheses after June 2010. Because of missing blood ion levels 478 patients operated upon in the earlier years, when this type of monitoring was not performed, had to be excluded. The blood tests to evaluate ions levels were carried out according to the European recommendation for the monitoring of MoM THAs [24]. For the final analysis 430 patients with 526 THAs were included.

### Exposure

Exposure of interest was all THAs with either a small (≤ 36 mm) or large (> 36 mm) head MoM bearing performed between 11/1998 and 03/2011 with ion blood levels. For the purpose of the study cobalt levels were taken for analysis.

### Outcomes

The primary aim of the study was to describe the pattern(s) of blood cobalt levels over time, assessed at least once and up to three times per THA at various time points during the follow up period, including after removal of the prosthesis (between 2010 and end of follow up, 31 December 2022). The cut off for blood cobalt levels to be considered as elevated was set at ≥ 2ug/l in accordance with the Scientific Committee on Emerging and Newly Identified Health Risks recommendations [5].

Secondary aims included the evaluation of predictors for elevated blood cobalt levels, and the assessment of an association between cobalt levels and clinical scores measured over the follow up period (from surgery to end of follow up, 31 December 2022): WOMAC pain and function, Harris Hip Score total, Harris Hip sub-scores limping and pain.

### Data collection

All pre-, per- and postoperative data was collected as part of the routine follow up of the arthroplasty cohort. The setup of the hospital-based arthroplasty registry has been described in previous studies [25].

### Statistics

We report continuous variables as mean (± standard deviation) or in cases of non-normal distribution, median and interquartile range. We checked normality of distribution graphically and according to the Shapiro–Wilk test. We report categorical variables as frequency and percentage.

Descriptive statistics were performed to report baseline characteristics for all the patients as well as for the two groups of interest, i.e. small (≤ 36 mm) vs. large (> 36 mm) head MoM THAs. Differences between the two groups at baseline were evaluated with Brunner-Munzel tests for continuous variables (a robust non-parametric test) and chi-square tests for categorical variables (or exact Fisher tests when cell frequencies < 5).

To assess the pattern of blood cobalt levels over time, we ran a mixed-effects model on log-transformed blood cobalt levels of MoM THA with at least two measurements before revision, with head size (small vs. large), time (as a categorical variable including two time points), and an interaction term as fixed effects, and THA as random intercept. To consider heteroscedasticity, we specified a variance–covariance matrix allowing different variances per stratum. Moreover, we ran a similar model with time as a continuous variable (years since operation), which additionally included a quadratic contrast for time effect. We checked normality of residuals graphically.

Due to limited sample size, only descriptive statistics were performed to report blood cobalt levels before and after revision according to head size.

Potential predictors of elevated cobalt levels (≥ 2ug/l) were evaluated using simple and multiple logistic regression models for small and large heads separately. We used the first available cobalt measure ≥ five years after operation to be after the running-in period. The following preoperative predictors were included: age, sex, Body Mass Index (BMI), smoking status (never vs. ever-smoker), and comorbidity count (beside osteoarthritis, classified as < 4 or ≥ 4 based on a former study [26]; only for small heads because the number of events was too low for large heads to include this variable in the model). Moreover, cup inclination (classified as ≤ 40° or > 40°) was included as operative predictor. Assumptions for the multiple regressions were met: independence of errors (Durbin Watson statistics = 2.03 and 1.49, respectively), no multicollinearity issue (variance inflation factor scores between 0.1 and 10), no outliers’ issue (Cook’s distances < 1), and satisfactory linearity of continuous predictive variables and log odds of the dependent variable.

To assess the relationship between cobalt levels and clinical outcomes (WOMAC pain and function scores, and Harris hip and limp scores), we included cobalt levels and outcome measurements that were assessed within the same year. We computed spearman correlations between cobalt levels and each outcome for all follow-up controls together.

Statistical significance was assessed at a two-sided 0.05 level. We performed all analyses using R Statistical Software (version 4.4.2; R Core Team, 2021).

## Results

Baseline characteristics of the 526 MoM THAs are reported in Table 1. The median age of the cohort was 59 years (interquartile range (IQR) 51–66) and 43.7% were females. Of the THA included, 427 (81.2%) were small head and 99 (18.8%) were large head THAs. The small head group included older patients than the large head group (median age 61 vs. 48 years, *p* < 0.001) and more females (46.6% vs. 31.3%, *p* = 0.006). Mean time to serology for metal ion levels in the cohort was 10.5 years (range 1.7—21.3 years). Revision for any reason was performed in 110 of these patients.

**Table 1:** Baseline characteristics of MoM THAs with comparison between small and large heads

	Total (*N* = 526)	Small head (≤ 36 mm) (*n* = 427, 81.2%)	Large head (> 36 mm) (*n* = 99, 18.8%)	*p*-value
**Woman (%)**	230 (43.7%)	199 (46.6%)	31 (31.3%)	0.006
**Age, median (IQR)**	59 (51–66)	61 (55–67)	48 (43.5–56)	< 0.001
**Age (%)**				< 0.001^1^
< 55 yrs	171 (32.5%)	106 (24.8%)	65 (65.7%)	
55 to 74.9 yrs	327 (62.2%)	293 (68.6%)	34 (34.3%)	
≥ 75 yrs	28 (5.3%)	28 (6.6%)	0	
**BMI, median (IQR)**	26.3 (23.5–29.5)	26.6 (23.7–29.8)	25.6 (23.1–27.8)	0.032
**BMI ≥ 30 (%)**	124 (23.6%)	105 (24.6)	19 (19.2)	0.254
**ASA**				0.030^1^
1–2	489 (93.0%)	392 (91.8%)	97 (98.0%)	
3–4	37 (7.0%)	35 (8.2%)	2 (2.0%)	
**Comorbidity count (%)**				0.145^1^
0–3	506 (96.2%)	408 (95.6%)	98 (99.0%)	
≥ 4	20 (3.8%)	19 (4.4%)	1 (1.0%)	
**Diabetes, yes (%)**	28 (5.3%)	26 (6.1%)	2 (2.0%)	0.136^1^
**Smoking never (%)**	261 (51.2%)	210 (51.0%)	51 (52.0%)	0.849
**Primary OA (%)**	373 (70.9%)	310 (72.6%)	63 (63.6%)	0.086
**Stem fixation cemented (%)**	305 (58.0%)	298 (70.0%)	7 (14.3%)	< 0.001
**Charnley**				0.254
A	197 (37.5%)	156 (36.5%)	41 (41.8%)	
B	213 (40.5%)	174 (40.7%)	39 (39.8%)	
C	115 (21.9%)	97 (22.7%)	18 (18.4%)	
**WOMAC pain, median (IQR)**	40 (30–50)	40 (30–50)	40 (25–50)	0.978
**WOMAC function, median (IQR)**	39.3 (25–50)	39.3 (27.7–50)	33.9 (25–50)	0.334
**SF-12 Physical Component, median (IQR)**	32.8 (27.6–38.5)	33.0 (27.5–38.2)	32.0 (27.6–39.6)	0.813
**SF-12 Mental Component, median (IQR)**	44.8 (34.5–55.1)	44.7 (35.1–55.4)	44.8 (32–53.6)	0.523

Missing values on smoking status (small head: 1, large head: 15), Charnley status (small head: 1), WOMAC pain (small head: 131, large head: 23), WOMAC function (small head: 135, large head: 23), and SF-12 Physical and Mental Component (small head: 136, large head: 25). ^1^Exact Fisher

**Fig. 1:**
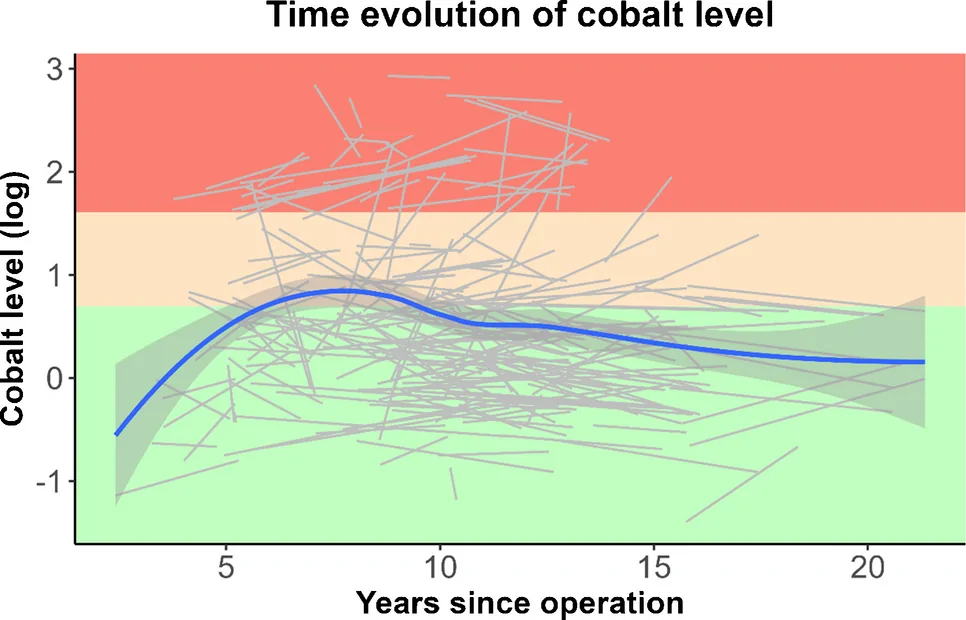
Time evolution of log-transformed cobalt level since operation. The green area includes cobalt values < 2ug/l. The orange area includes cobalt values ≥ 2ug/l and ≤ 5ug/l. The red area includes cobalt values > 5ug/l. The blue line shows locally weighted smoothing with 95% confidence interval

**Fig. 2:**
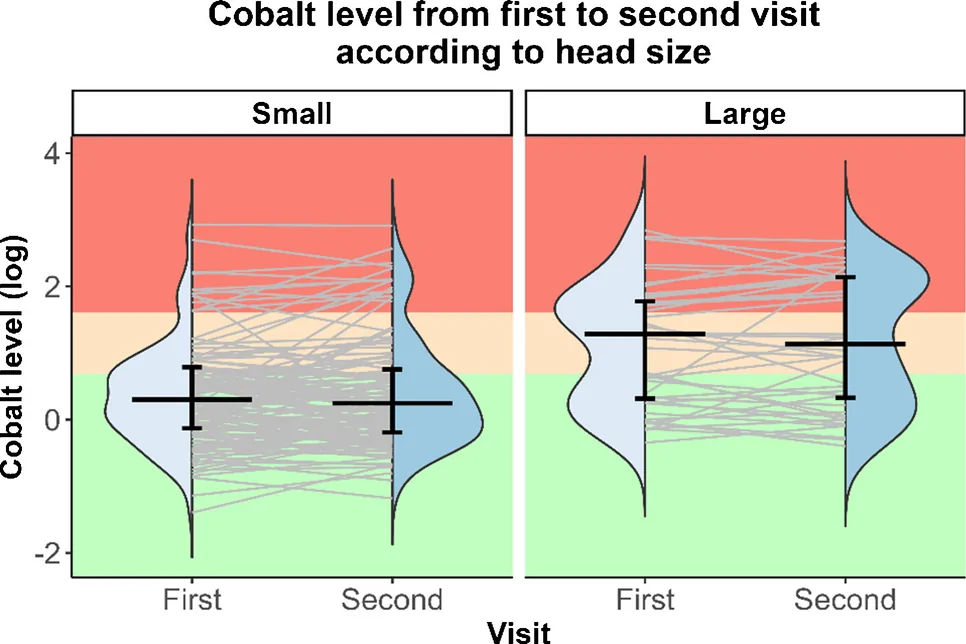
Log-transformed cobalt level from first to second visit according to head size. The green area includes cobalt values < 2ug/l. The orange area includes cobalt values ≥ 2ug/l and ≤ 5ug/l. The red area includes cobalt values > 5ug/l. Crossbars and error bars are medians and interquartile ranges, respectively

The pattern of blood cobalt levels over time considering patients with at least two serologies before revision (*n* = 205, 157 small heads, 48 large heads) is shown in Fig. 1 (in years since operation) and Fig. 2 (for the two consecutive serologies according to head size). The mixed-effects model revealed that cobalt ion levels were higher for patients with large compared to small heads (non-transformed medians ± interquartile range: 3.5 ± 5.3 vs. 1.3 ± 1.4, *p* < 0.001). However, we did not find any effect of time on cobalt ion levels between the two consecutive serologies (*p* > 0.52) or when using time as a continuous variable (*p* > 0.21; quadratic contrast *p* > 0.22). Moreover, the interaction between head size and time was not significant (*p*-values > 0.42). The findings were similar after including only unilateral THAs.

**Fig. 3:**
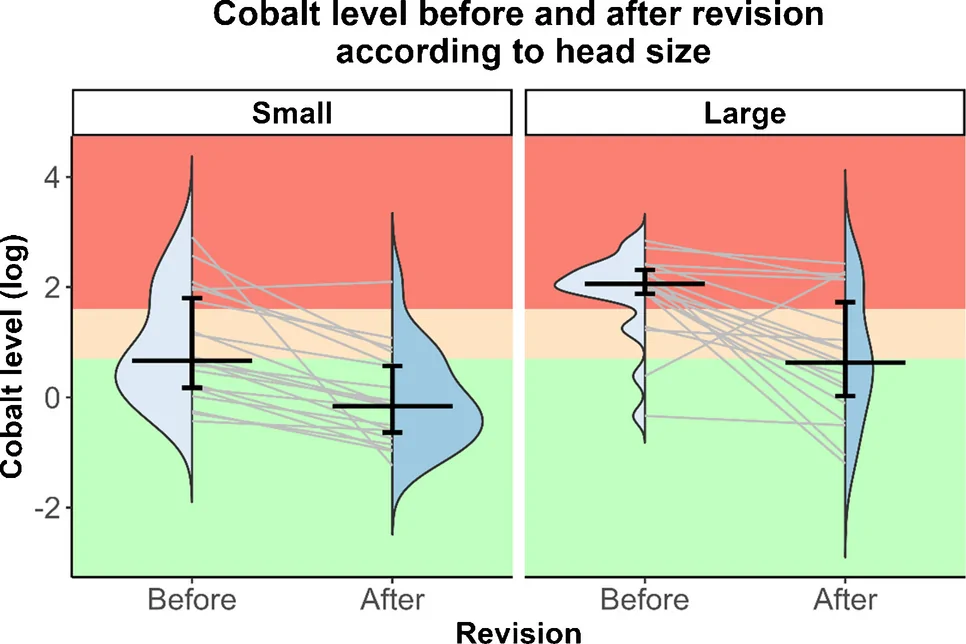
Log-transformed cobalt level before and after revision for small and large MoM THAs. The green area includes cobalt values < 2ug/l. The orange area includes cobalt values ≥ 2ug/l and ≤ 5ug/l. The red area includes cobalt values > 5ug/l. Crossbars and error bars are medians and interquartile ranges, respectively

Cobalt ion levels before and after MoM prosthesis removal for large (*n* = 19, non-transformed medians ± IQR = 7.8 ± 3.8 vs. 1.9 ± 6.3) and small heads (*n* = 20, non-transformed medians ± IQR = 1.9 ± 4.9 vs. 0.9 ± 1.2) indicated a reduction to values in the normal range for large heads as shown in Fig. 3. Some patients still showed elevated cobalt ion levels after revision (15.4% overall, 26.3% for large head, and 5.0% for small heads). Post-revision measures were taken at a median time of 16.7 weeks (IQR 42.1) after revision.

Regarding predictors of elevated cobalt levels for small head MoM THAs, we found that never smoker patients were more likely to show elevated cobalt levels than ever smoker patients (unadjusted OR = 1.77, 95%CI [1.03 to 3.04], *p* = 0.037) in the univariate analysis. However, after adjustment never smokers were no longer associated with elevated cobalt levels (*p* = 0.120) (Table 2). For large heads, we found that women were more likely to show elevated cobalt levels than men (adjusted OR = 3.56, 95%CI 1.17–10.84, *p* = 0.02). Other predictors were not significant.

**Table 2:** Descriptives, simple and multivariable logistic regression models investigating variables associated with elevated cobalt levels (> 2ug/l) for small and large heads

	Descriptives		Simple logistic regression		Multivariable logistic regression	
	Cobalt levels ≤ 2	Cobalt levels > 2	Odds ratio [95%CI]	*p*-value	Adjusted odds ratio [95%CI]	*p*-value
***Small heads***						
** Age at operation, median (IQR)**	62 (55–67.5)	64 (57–69)	1.02 [0.99 to 1.05]	0.146	1.01 [0.99 to 1.04]	0.324
** Female sex**	96 (43.0%)	38 (52.1%)	1.44 [0.85 to 2.44]	0.181	1.23 [0.70 to 2.16]	0.473
** BMI ≥ 30**	169 (75.8%)	50 (68.5%)	1.44 [0.81 to 2.57]	0.219	1.34 [0.74 to 2.45]	0.337
** Never smoker**	106 (47.5%)	45 (61.6%)	1.77 [1.03 to 3.04]	**0.037**^**+**^	1.59 [0.89 to 2.85]	**0.120**^**+**^
** Cup inclination ≤ 40**	96 (43.0%)	37 (50.7%)	1.36 [0.80 to 2.31]	0.256	1.41 [0.82 to 2.42]	0.214
** Comorbidities > 4**	7 (3.1%)	5 (6.8%)	2.27 [0.70 to 7.38]	0.173	2.19 [0.65 to 7.43]	0.208
***Large heads***						
** Age at operation, median (IQR)**	48.5 (44–55.25)	51 (44–57)	1.01 [0.97 to 1.06]	0.547	1.02 [0.97 to 1.07]	0.514
** Female sex**	6 (18.8%)	18 (43.9%)	3.39 [1.15 to 10.00]	0.027*	3.56 [1.17 to 10.84]	0.025*
** BMI ≥ 30**	26 (81.2%)	32 (78.0%)	1.22 [0.38 to 3.87]	0.737	1.21 [0.35 to 4.17]	0.761
** Never smoker**	14 (43.8%)	21 (51.2%)	1.35 [0.53 to 3.42]	0.527	1.04 [0.38 to 2.84]	0.940
** Cup inclination ≤ 40**	8 (25.0%)	7 (17.1%)	0.62 [0.20 to 1.93]	0.408	0.62 [0.18 to 2.10]	0.442

*p < 0.05, ^+^*p* < 0.15, *n* = 296 for small heads and 73 for large heads. *IQR* interquartile range

Considering THAs with a clinical visit and a serology obtained within one year (*n* = 361), we did not find any significant correlation between cobalt levels and clinical outcomes (WOMAC pain and function scores, and Harris hip and limp scores; Spearman’s r values < 0.08, *p*-values > 0.17). The findings were similar when including only small heads THAs.

## Discussion

Our results showed that cobalt levels did not vary over time, in large and small head MoM THAs. Except for higher cobalt levels in women with large head MoMs, the results did not reveal any predictive factor(s) for the elevation of cobalt levels. Moreover, no association between cobalt levels and clinical outcomes was found in large or small head MoM THAs.

In vitro studies have demonstrated a running-in phase after implantation of a THA with MoM bearing [12]. Clinical studies reported different ion level patterns after MoM THA. Two studies on resurfacing implants showed that after this running-in phase, ion levels remained stable within two years or even decreased [15, 16], whereas other studies showed that ion levels increased steadily. Studies showing a constant increase had small sample sizes ranging from six to 71, and they included mainly large head THAs and resurfacing [13, 14]. One study on 56 resurfacing hips showed a second cobalt level increase at ten years follow up [17]. Bernstein et al. [27] assessed cobalt patterns in 87 small head MoM THAs and observed a peak of blood cobalt levels (2.87ug/l) at four years followed by a decrease at nine years (2.0ug/l) [28]. In a small series including 20 MoM THA with 28 mm head, the median cobalt level remained stable at 21 years postoperatively (median value 1.04µh/). Our results are consistent with this study. We included a larger number of THAs; among the 205 MoM THAs with at least two cobalt measurements, cobalt levels remained constant over time, even after stratification by head size. This element is an important finding for the follow up of patients with MoM THA. Indeed, published guidelines for monitoring patients with MoM THA state that regular dosages of ion levels should be carried out [4]. However, taking these blood tests is burdensome for patients and increases health costs [11]. Reito et al. [29] already recommended less frequent blood measurements for hip resurfacing with initial blood values below the thresholds. As part of our regular follow-up and based on these findings, we have decided to go further by discontinuing regular blood tests for metal ion levels in patients with MoM THA. On the other hand, if patients were to present symptoms at the level of their hip arthroplasty, at that time as part of the pain investigation assessment, a dosage of the ion level is carried out.

Normal blood cobalt values range from 0.1 to 1.21 µg/l [30], but there is no consensus for a threshold for blood cobalt levels considered as elevated [5, 31]. In a recent systematic review including 981 MoM THAs of all head sizes the mean serum cobalt concentration was 3.5 ± 5.1 µg/l [32]. After revision of MoM THA with change of bearing surface to a non-MoM bearing, we observed a decrease in cobalt levels to levels considered normal, for the majority of small and large heads MoM. Other studies including only large-headed MoM THAs observed similar results to ours, after revision surgery of large head MoM THAs a maximum decrease in ion blood levels occurred within the first two months and up to one year after revision surgery [33–35]. Decrease in blood metal ion levels was also observed both after isolated cup MoM and total THA revision [36]. These results are reassuring for both patients and their health care providers.

Previous studies already showed that women were more likely to have elevated cobalt levels [20, 21] and this is confirmed in our study. Another factor reported in the literature as associated with elevated blood metal levels in large MoM THAs, is cup inclination [18, 19], which was not evaluated in our study.

Studies including large (≥ 36 mm) head MoM THAs or hip resurfacing [37] with sample sizes ranging from 76 to 383 did not observe a correlation between ion levels and clinical scores, assessed by the Oxford Hip Score, HHS, WOMAC, SF-26 and/or UCLA [22, 23, 38]. Our results are in accordance with these previously published studies, although these studies did not include small head MoM THAs. The relation between blood metal ion levels and clinical outcomes after small MoM THA remains little studied. In a recent retrospective study including 61 patients (without information on head size), no correlation between cobalt levels and Oxford Hip Scores was reported [39]. Clinical outcome assessment is important in decision-making regarding the management of patients with MoM THA. However, guidelines for surveillance of these patients do not include patient reported outcome measure thresholds [8]. To help decision-making regarding the care of MoM THA, a risk score was developed, including the Harris Hip Score and blood ion levels; both of these variables were found to be predictive factors for revision of a MoM THA [40].

Our study has some limitations. First because of missing cobalt blood levels for the patients operated in the earlier years, over 50% of THAs meeting the selection criteria had to be excluded. However, our cohort included a large number of MoM THAs compared to previous published studies, and the baseline characteristics of the excluded THAs remained similar to the included cohort. Second, cobalt has a renal metabolism, and renal failure might be a factor favouring cobalt level increase. However, we did not assess renal function of the included patients. At baseline less than 4% of the cohort had four or more comorbidities. Given that the prevalence of renal failure [41] is less than for high blood pressure or hypercholesterolaemia [42] in the general population of our country, the few potential cases of renal failure should not have influenced our results.

## Conclusion

We observed stable cobalt levels over time in small and large MoM THAs. We found one risk factor for elevated cobalt levels in large MOM THAs, and clinical outcomes were not associated with cobalt levels. Repeat laboratory workups in small and large head MoM THAs seem unnecessary given that the cobalt values remained stable. Removal of the MoM prosthesis largely reduced cobalt concentrations to nearly normal levels in the months after the intervention.

## Data Availability

No datasets were generated or analysed during the current study.
